# How the delivery of HIV care in Canada aligns with the Chronic Care Model: A qualitative study

**DOI:** 10.1371/journal.pone.0220516

**Published:** 2019-07-26

**Authors:** Clare Liddy, Esther S. Shoemaker, Lois Crowe, Lisa M. Boucher, Sean B. Rourke, Ron Rosenes, Christine Bibeau, Claire E. Kendall

**Affiliations:** 1 C.T. Lamont Primary Health Care Research Centre, Bruyère Research Institute, Ottawa, Ontario, Canada; 2 Department of Family Medicine, University of Ottawa, Ottawa, Ontario, Canada; 3 ICES, Toronto, Ontario, Canada; 4 Ottawa Hospital Research Institute, Ottawa, ON, Canada; 5 Department of Psychiatry, University of Toronto, Toronto, Ontario; 6 Li Ka Shing Knowledge Institute, St. Michael’s Hospital, Toronto, Ontario; Hofstra University, UNITED STATES

## Abstract

With the advent of continuous antiretroviral therapy, HIV has become a complex chronic, rather than acute, condition. The Chronic Care Model (CCM) provides an integrated approach to the delivery of care for people with chronic conditions that could therefore be applied to the delivery of care for people living with HIV. Our objective was to assess the alignment of HIV care settings with the CCM. We conducted a mixed methods study to explore structures, organization and care processes of Canadian HIV care settings. The quantitative results of phase one are published elsewhere. For phase two, we conducted semi-structured interviews with key informants from 12 HIV care settings across Canada. Irrespective of composition of the care setting or its location, HIV care in Canada is well aligned with several components of the CCM, most prominently in the areas of linkage to community resources and delivery system design with inter-professional team-based care. We propose the need for improvements in the availability of electronic clinical information systems and self-management support services to support better care delivery and health outcomes among people living with HIV in Canada.

## Introduction

As people with HIV age, their healthcare becomes increasingly complex due to co-morbidities associated with aging and neurocognitive disorders related to HIV and antiretroviral therapy (ART) [1–5]. In the early ART era, studies using disease-specific indicators showed that HIV specialists and those with larger HIV caseloads provided a higher quality of care than generalist providers[6–10]. Today, it is increasingly recognized that HIV specialists may be less proficient than primary care providers in providing preventative care [11–14] and in managing co-morbidities such as diabetes and hyperlipidemia [11,15–20]. Thus, there are calls for new models of care that can comprehensively attend to the diverse physical, mental, and social needs of people living with HIV [21–23].

Patient-centered care, in which patients are involved in individualized care that respects and responds to their preferences [24,25], has been recognized to be important for patients with multimorbidities [26], particularly people living with HIV [27]. Central to this approach is the emphasis it places on the partnership between patient and provider, and the facilitation of the active involvement of patients in their own care[26]. The Chronic Care Model (CCM) developed by Wagner and colleagues [28] identifies six elements essential to optimizing patient-centered care for people with chronic conditions: healthcare organization, self-management support, delivery system design, decision support, clinical information systems, and community linkages (see Table 1 for an overview of the CCM elements). Taken collectively, these elements are intended to produce effective interactions between *proactive prepared* practice teams and *informed activated* patients. Since HIV has increasingly been recognized as a chronic condition, there is growing interest in applying the CCM model to improve functional and clinical outcomes [29,30]. However, little is known about how HIV care settings presently incorporate the CCM approach to support care delivery for people living with HIV. In this study, we used semi-structured interviews with key informants from Canadian HIV care settings to examine the extent to which HIV care settings incorporate the six essential elements of the CCM.

**Table 1:** Elements of the Chronic Care Model [31].

**Health system—Organization of healthcare**	• Emphasizes the need for organizational goals to prioritize chronic care. • Requires strong leadership that exhibits traits such as creativity, compassion and advocacy, focuses on optimizing care delivery, and ensures patient-centeredness. • The set-up of the financial structure including remuneration and relationships with purchasers and insurers is integral to the CCM and must recognize and reward high quality chronic care.
**Self-management support**	• It is critical for people living with chronic conditions to receive support to manage their own care, and to be positioned as equal collaborators in their care.
**Delivery system design**	• Highlights the need for team-based care, with interdisciplinary team members having clear roles in proactively optimizing patient visits.
**Decision support**	• Ensures the integration of evidence-based guidelines into daily clinical practice. • Facilitates access to specialist expertise, specifically within primary care settings.
**Clinical information systems**	• Computerized systems can be used to implement decision support strategies, provide quality metrics back to physicians, and create clinical registries for management of patient populations.
**Community resources and policies**	• Linkages with community-based resources are necessary to enhance care for people with chronic conditions.

## Methods

The Ottawa Health Sciences Network Research Ethics Board (protocol #20140649-01H) and the Bruyère Continuing Care Research Ethics Board (protocol #M16-15-011) approved the study. Survey participants from Phase 1 were gave written consent to be contacted for a follow-up interview. At the beginning of the interviews, oral consent was obtained.

### Study design

We conducted a sequential explanatory mixed methods study involving three phases: 1) a quantitative phase employing a survey of Canadian HIV care settings, 2) a qualitative phase employing semi-structured interviews with key informants, and 3) an integration phase in which interviews were used to contextualize and aid interpretation of the survey results. In phase one of the study [32], we identified organizational patterns and gaps in the delivery of HIV care in Canada. The results were used to develop the semi-structured interview guide, pilot tested internally with the clinicians and people with lived experience who are members of the research team, and refined iteratively. This paper focuses on phase two, and uses directed content analysis guided by the elements of the CCM.

### Theoretical framework

We applied the CCM as a framework for understanding how the principles of patient-centered chronic disease management and prevention can help advance primary healthcare for people living with HIV in Canada.

### Setting

This study is part of a larger Canadian Institutes of Health Research funded program of research taking place in Ontario, Manitoba, and Newfoundland and Labrador (https://www.lhiv.ca/).

### Participants

Care settings that participated in the quantitative survey in phase 1 were invited to participate in a telephone interview in the language of their choice (English or French) and are described in detail elsewhere [32]. An environmental scan and expert knowledge of team members was used to identify and recruit settings. Settings were included if they presented themselves as caring for people living with HIV. Each setting was asked to identify a team member with extensive knowledge of the care processes and structures of the setting as a key informant for the interview. To ensure a diverse range of viewpoints for this phase of the study, we targeted survey participants who varied in terms of position (e.g. provider, manager), type of setting, populations served, region, and the setting’s PCMH-A score. Participants were given a $75 gift card for their participation.

### Data collection

Individual semi-structured telephone interviews with key informants from the participating settings were conducted by two experienced qualitative researchers (DR, PhD in Exercise Sciences and NT, PhD candidate in Population Health) between November 2016 and February 2017. They lasted approximately 60 minutes and were audio recorded. Participants were asked questions about how their clinic provides patient-centered care and the services available to people with HIV (see S1 Appendix for interview guide).

### Data analysis

Interviews were transcribed verbatim and imported into NVivo® 11 for analysis [33]. Two coders with experience in qualitative research and from different academic disciplines (ES PhD in Population Health and JP MA in Psychology) coded the data independently using a thematic framework [34] based on the CCM. They examined the extent to which the interview data mapped onto the CCM’s six elements. The reviewers met weekly to identify any disconfirming elements, and regularly discussed the findings with the other team members, including two people living with HIV.

## Results

Twelve participants out of the 22 HIV care settings that participated in the first phase of the study, were interviewed. They were located in urban centers in five Canadian provinces: Saskatchewan (n = 1), Manitoba (n = 2), Ontario (n = 7), Quebec (n = 1), and New Brunswick (n = 1). Six participants came from hospital-based specialist care settings and six from primary care settings. Nine participants were clinicians (two infectious disease specialists, two family physicians, four registered nurses, one nurse practitioner) and three were managers/directors. A detailed description of the participating settings can be found elsewhere [32].

### Health system—Organization of healthcare

One participant provided an example of how involving patients in leadership facilitates high quality of care: *“Having patient and staff members that are on our board of directors helps to guide […] patient directed or patient involvement in program planning or services*, *service planning”* (P3, Medical Director/Family Physician). Another offered an example of a creative solution to a patient issue:

*We had one particular person who was pregnant at the time [and] had lost access to her financial supports*, *to her benefits*, *which then put her at risk for potentially losing access to her HIV meds*. *Not at a great time*, *when you’re pregnant*. *And so [we] put the call out and immediately got responses and people sent [leftover drugs] to our clinic*. *And we have a healthy baby girl for that*. (P9, Physician)

However, participants from hospital-based settings at times described a perceived risk between their values as HIV providers and those of the larger organization where hospital administrators do not necessarily understand the unique needs of people living with HIV. As one participant stated: *“If leaders knew what the challenges were in providing HIV care*, *maybe they*, *maybe would be better able to hear what we as frontline workers need”* (P4, Nurse Practitioner). None of the participants mentioned the financial structure of their organization to impact the care they were able to provide.

### Self-management support

Many participants reported offering some components of self-management support, from in-house education to linkages with community-based programs, though others reported no such support. Overall, settings offered few formal self-management programs to improve patients’ knowledge, skills and confidence in managing their conditions, especially with respect to HIV. One participant identified the need to further integrate self-management into their care processes: *“I think we still have a ways to go in terms of looking at HIV as a chronic disease and really encouraging self-management*” (P10, Program Manager).

### Delivery system design

Most HIV settings in this study provided team-based care, with multiple different allied health providers on site, including pharmacists, dietitians, social workers, and addiction counsellors. One participant noted that *“our clinic is very multidisciplinary and it’s a team approach*. *So just depending on the patient and where they’re at*, *they have access to a range of healthcare providers”* (P10, Program Manager). Another outlined how their services extend far beyond the medical care needs of their patients: “*It’s a dialogue between nurse*, *patient and doctor and then around the services*. *[*…*] We even opened a legal clinic in collaboration with lawyers in order to offer our patients living with HIV services if they do have- experience problems due to their HIV status”* (P11, Acting Director). Other participants stated that even when providers are not seeing a patient simultaneously, they support each other in their roles and the patients in their care, for example, through case conferences and team meetings.

### Decision support

Although clinical decision support was not frequently mentioned, most participants embraced patient decision support to optimize care, and promoted means to ensure patients are supported in making care decisions. Several participants noted that they link with other specialties when relevant, particularly for patients struggling with mental health. Participants described several ways they use computerized systems to aid decision making and optimize care for people living with HIV: *“We generate reports from our EMR*. *They come to the nurse and physician and we were running those meetings with social work and outreach workers present to review sort of the most at-risk cases and to try and make some management plans to support someone to get into care on treatment*” (P3, Medical Director/Family Physician). One clinical decision support strategy that was mentioned was the use of “triggers” in patients’ charts: *“We’ll have triggers in their templated notes to remind people about some of the services that are available…more of a proactive as opposed to a reactive approach*” (P3, Medical Director/Family Physician). Participants also described novel ways of ensuring patients received access to HIV-specific expertise, such as video-conferencing, but one participant noted some providers are hesitant to adopt such technologies.

### Clinical information systems

One study participant identified the need to improve current decision support systems, which was directly related to the absence of electronic information systems: *“How do we work better with our electronic systems to track the people who are lost to follow up*? *It would be great to be able to generate those lists of people that we haven’t seen in over a year*. *Some of them may have died*. *We may not even know*, *which is very upsetting”* (P4, Nurse practitioner). In addition, several participants expressed frustration at their settings’ lack of adequate clinical information systems, noting that this left them inadequately resourced to care for their population of patients: “*We don’t have any statistics*, *[*…*] you can’t [*…*] in just one click*, *give me the most recent HIV patients I’ve seen in the last year*, *in the last six months*. *How many are taking this medication*, *how many got a flu shot*, *how many are due for a pap test*?*”* (P12, Specialist).

### Community resources and policies

Many participants described community services as mechanisms to address clinical service gaps: *“So we can’t offer that long-term access [to mental health services]*. *We will try to access counselling or support services throughout community if we can”* (P9, Physician). One participant outlined the importance of connecting patients with services in their community:

*[W]e have direct access to translation services through the health region [and] there are community-based organizations we would work in partnership with too*. *So I’m just thinking about one patient [*…*] who was from Thailand and was having quite a few [*…*] language challenges*. *So we ended up working with him and then an interpreter through this community-based organization*. (P10, Program Manager).

Many participants described how they facilitated linkages with community resources that have the potential to improve patients’ living conditions beyond their health: “*Let’s say you have HIV*, *it’s stable*, *but you’re having surgery on your knee*, *a knee replacement*, *‘cause you’re aging*. *Who’s going to help you*? *So you can get Food for Life for those three weeks after surgery”* (P4, Nurse Practitioner). Some participants described formalizing these community linkages through partnership agreements, while all participants ensured their clients are linked with appropriate community services.

## Discussion

To our knowledge, our paper is the first to provide an in-depth, comprehensive view of HIV care delivery through the lens of the CCM, making our results of interest to practitioners, policy makers and people with lived experience aiming to optimize care delivery for a diverse and aging population of people living with HIV.

We found most settings delivered care in concordance with many components of the CCM, regardless of whether the setting was specialty focused or embedded in HIV primary care. In particular, settings’ efforts to offer collaborative and inter-professional team-based care and to build connections between clients and available community resources are well aligned with the CCM. However, organization of healthcare and self-management support varied substantially between settings and were often lacking. This may reflect the fact that such programs are still not well integrated into healthcare settings overall, as well as the lack of targeting of such programs specifically for people living with HIV. In addition, initiatives undertaken in the HIV community, including peer support, may not be explicitly labeled as self-management support or may operate more informally, yet may contribute to improving self-management skills. Identifying and improving linkage to resources in the community that provide self-management support may help to address this gap. A number of studies have in fact shown an association between a setting’s use of clinical information systems and improved chronic disease self-management support [35–38]. Participants in this study described their need for improved electronic systems to better care for their clients.

Guided by the CCM, Canadian provinces have invested in developing new models of primary care. Settings’ organizational characteristics generally reflect components of the CCM (e.g. population approach, delivery system design, clinical information systems), but models vary substantially [39]. Measuring the specific organizational features associated with improved care is essential to understanding quality of chronic care delivery [40]. Certain CCM elements such as delivery system design [41], decision support, clinical information systems [42], and self-management support [37] have been shown to improve some aspects of HIV care in a similar way to care for other conditions [37], but those studies were heterogeneous, often population-specific, and lacked sufficient detail to direct policy. While the CCM provides a guiding framework for the shift in HIV care from a specialist model to a primary chronic care approach, research is required to understand the association of each element of the CCM in improving care for people with HIV.

Our results build on previous research that has highlighted the growing needs of people living with HIV and a delivery approach that can encompass the CCM [29,42,43]. Care for chronic health problems requires coordination across professional and disciplinary boundaries to promote patient-centered care [38]. A handful of studies have explored how best to support a primary care approach to HIV care delivery that allows timely and continuous access to specialist expertise. Promising practices include the inter-professional provision of care by new types of allied health professionals [44], co-management [13], community-based rather than hospital-based care [45], and the use of telephone consultants [46]. However, in many regions of Canada, these strategies, which rely chiefly on co-location and synchronous communication between physicians and allied providers, are impeded by small numbers of HIV specialists, long wait times, geographic barriers, lack of specialized equipment (e.g. telemedicine), and outstanding privacy concerns.

### Limitations

This study has several limitations. We cannot be certain that the views expressed by the key informants of participating care settings reflect the views of other people working in those settings. Some provinces are well represented among the responding clinics whereas clinics in other provinces did not respond to our request for participation. HIV settings from rural and remote areas are also not well represented, likely because of lack of HIV-specific services in these settings [47]. Finally, our study only looked at HIV care settings in Canada. HIV care settings in other countries such as the Ryan White clinics in the United States, with differing care organization and financing mechanisms, may be adapting to the Chronic Care Model in quite different ways. However, few studies to date have addressed this issue [48–50].

## Conclusion

We were able to outline the components of HIV care settings in Canada that align with the CCM, specifically in the areas of inter-professional team based care and linkage to community resources. Identifying theoretically grounded care approaches is directly related to improving the applicability of research evidence to care practices [51]. We note a need for improvement in the availability of electronic clinical information system and self-management support services in some settings to improve care delivery and health outcomes of people living with HIV. In supporting a CCM care approach, these findings are especially timely, given that people living with HIV are aging and their health needs are becoming more complex, thus requiring responsive, patient-centered healthcare systems.

## Supporting information

S1 Checklist COREQ checklist. (DOCX)

S1 Appendix Appendix A. Interview guide.
(DOCX)
