# Supplementary material for: How the delivery of HIV care in Canada aligns with the Chronic Care Model: A qualitative study
Source: PLoS One. 2019 Jul 26;14(7):e0220516. doi: 10.1371/journal.pone.0220516 (PMC6660092; doi:10.1371/journal.pone.0220516)
Supplement: S1 Appendix — Appendix A. Interview guide. (DOCX) [file pone.0220516.s002.docx]

**Appendix A**

Interview Guide

1. Can you tell me a little bit more about your role in the clinic? How active are you in providing care to persons living with HIV?
2. The on-line survey we conducted asked a number of questions about your clinic’s ability to provide patient-centred care. We know that patient-centred care means different things to different people, and we are interested in how you would describe what patient-centred care means to you?
3. For patients, it might mean that they are treated as a whole person, and that the care they receive reflects their values and preferences.
4. Clinicians may view patient-centredness as advocating for their patients to ensure their health care needs are met.
5. Clinics and health care organizations might describe themselves as providing patient-centred care when they include patients in their decision-making, and use the results of patient surveys to inform their decisions.
6. Policy makers sometimes describe patient-centredness as a measure of health system performance or as a principle to strive for when designing health services within the budgets they have been allocated.
7. Public health practitioners may feel that patient-centredness should be less concerned with meeting the desires of individual patients and more concerned with addressing the social determinants of health, which they argue would prevent many citizens from becoming patients in the first place.

Source: http://healthydebate.ca/opinions/patient-centred-mean-achievable

1. Can you tell us about how your clinic approaches patient-centred care? Would you mind sharing some examples?
2. A patient with stable HIV comes into your clinic to meet with a physician or team member. Can you describe what services are available? What are some of the strategies you and your team use to help your patients access necessary services? Can you share some examples?
   1. How do you ensure they have access to up-to-date pharmacy advice, or provide support for acquiring medications that are too expensive for them?
   2. How do you link them with primary health care/specialty care for their other co-morbid conditions, such as diabetes or other chronic health disease?
   3. How would they access mental health services, or peer counselling support programs?
   4. Do you offer any on-site or access to community-based chronic disease self-management programs?
   5. How do you support sub-populations of people with HIV (i.e. low-income, women, refugees/immigrants), to navigate the different services?
   6. Do you have any specific protocols in place to support patient privacy, particularly for someone living with HIV, given the significant stigma attached to this disease?
3. What is the role of senior managers or leaders in building effective team-functioning in your clinic?
4. How are care/administrative decisions made in your clinic?
5. If these leaders were more engaged on a day-to-day basis, how might this affect functioning in the clinic?
6. How could the role of management be improved?
7. If you could set up a patient-centred care system for people living with stable HIV, what would it look like? How would your clinic need to change?
